# PPARG stimulation restored lung mRNA expression of core clock, inflammation‐ and metabolism‐related genes disrupted by reversed feeding in male mice

**DOI:** 10.14814/phy2.15823

**Published:** 2023-09-13

**Authors:** Oksana Shlykova, Olga Izmailova, Alina Kabaliei, Vitalina Palchyk, Viktoriya Shynkevych, Igor Kaidashev

**Affiliations:** ^1^ Poltava State Medical University Poltava Ukraine

**Keywords:** core clock genes, inflammation, lung, pioglitazone, PPARG

## Abstract

The circadian rhythm system regulates lung function as well as local and systemic inflammations. The alteration of this rhythm might be induced by a change in the eating rhythm. Peroxisome proliferator‐activated receptor gamma (PPARG) is a key molecule involved in circadian rhythm regulation, lung functions, and metabolic processes. We described the effect of the PPARG agonist pioglitazone (PZ) on the diurnal mRNA expression profile of core circadian clock genes (*Arntl*, *Clock*, *Nr1d1*, *Cry1*, *Cry2*, *Per1*, and *Per2*) and metabolism‐ and inflammation‐related genes (*Nfe2l2*, *Pparg*, *Rela*, and *Cxcl5*) in the male murine lung disrupted by reversed feeding (RF). In mice, RF disrupted the diurnal expression pattern of core clock genes. It decreased *Nfe2l2* and *Pparg* and increased *Rela* and *Cxcl5* expression in lung tissue. There were elevated levels of IL‐6, TNF‐alpha, total cells, macrophages, and lymphocyte counts in bronchoalveolar lavage (BAL) with a significant increase in vascular congestion and cellular infiltrates in male mouse lung tissue. Administration of PZ regained the diurnal clock gene expression, increased *Nfe2l2* and *Pparg* expression, and reduced *Rela*, *Cxcl5* expression and IL‐6, TNF‐alpha, and cellularity in BAL. PZ administration at 7 p.m. was more efficient than at 7 a.m.

## 1 INTRODUCTION

The circadian rhythm regulates lung functions, including upper and lower airway capacity, respiratory volumes, mucus production alveolar capillary flow, and inflammatory cell flux in physiological conditions. Diurnal variation of the clinical signs, symptoms, and exacerbations also occurs in pulmonary diseases (Sundar, Yao, et al., 2015a). Physiological activity of lung function has a diurnal variation with the low value in the early morning and the upper value at noon. Usually worsening of respiratory symptoms occurs in patients with asthma or chronic bronchitis during the early morning (Barnes, 1985). Circadian expression of core clock genes described for lung tissues and its disorders influenced respiratory functions and all types of respiratory and inflammatory cells (Evans & Davidson, 2013; Gibbs et al., 2014; Pekovic‐Vaughan et al., 2014; Sundar, Ahmad, et al., 2015b). Despite our recent knowledge of how the lung circadian system influences lung physiology and pathophysiology, we need the development of new treatments for pulmonary diseases using instruments of chronobiology and chronopharmacology (Sundar, Yao, et al., 2015a).

Nutrients have a strong impact on circadian rhythm, influence metabolic processes in various cells and tissues, and affect the molecular clock gene expression (Oosterman et al., 2015). The alteration of the peripheral clock genes' rhythm might be induced by the changing of the eating rhythm. Night‐ and evening‐time eating had a deleterious impact on inflammatory markers, and provoked low‐grade systemic inflammation, high blood pressure, sleeping disorders, glucose intolerance, and weight gain (Carithers‐Thomas et al., 2010; Marinac et al., 2015; Mundula et al., 2022; Scheer et al., 2009).

The clock gene rhythm, low‐grade inflammation, immune response, and metabolism are closely related through the number of transcriptional factors and cellular pathways (Cox et al., 2022; Fagiani et al., 2022; Li, Liu, Meng, et al., 2022; Ray, 2022; Shirato & Sato, 2022). These include peroxisome proliferator‐activated receptor gamma (PPARG) and PPARG coactivator 1 alpha (PGC‐1 alpha) (Fedchenko et al., 2022; Hara et al., 2001; Vallée et al., 2019). PPARG is a key regulator of fetal lung maturation (Lee et al., 2020), lung immunity (Nobs & Kopf, 2018), fibrosis (Deng et al., 2012), vasculature (Hart, 2008), and metabolism (Kökény et al., 2021), etc. PPARG is a promising target in lung pathologies such as chronic airway inflammation (Belvisi et al., 2006), asthma and COPD (Al Sharif, 2021; Byelan et al., 2017; Rogliani et al., 2018; Tseng, 2022), pulmonary vascular disease (Afdal & AbdelMassih, 2018), pulmonary artery hypertension (Hansmann et al., 2020), fibrosis (Milam et al., 2008), and cancer (Li et al., 2006). Activation of PPARG is a possible tool for the realization of pleiotropic effects. Such influence might be maintained by 2,4‐thiazolidinediones (glitazone) and its derivate with a plethora of pharmacological activities (Kajal et al., 2022).

One of the most investigated PPARG agonists is pioglitazone (PZ). PZ increased PPARG expression and inhibits local and systemic inflammation during acute lung injury (Editorial Office, 2022). PZ attenuates a dysfunction of alveolar macrophages (Yeligar et al., 2021), ameliorates COPD‐induced endothelial dysfunction (Abdelhafez et al., 2021), prevents obesity‐related airway hyperreactivity (Proskocil et al., 2021), decreases airway remodeling in OVA‐induced inflammation (Meng et al., 2018), and reverses pulmonary hypertension (Legchenko et al., 2018).

The study assessed the effects of reversed feeding (RF) and PZ on the expression of the core clock, inflammatory and metabolic genes in the male murine lung.

## 2 MATERIALS AND METHODS

### 2.1 Animals

**FIGURE 1:**
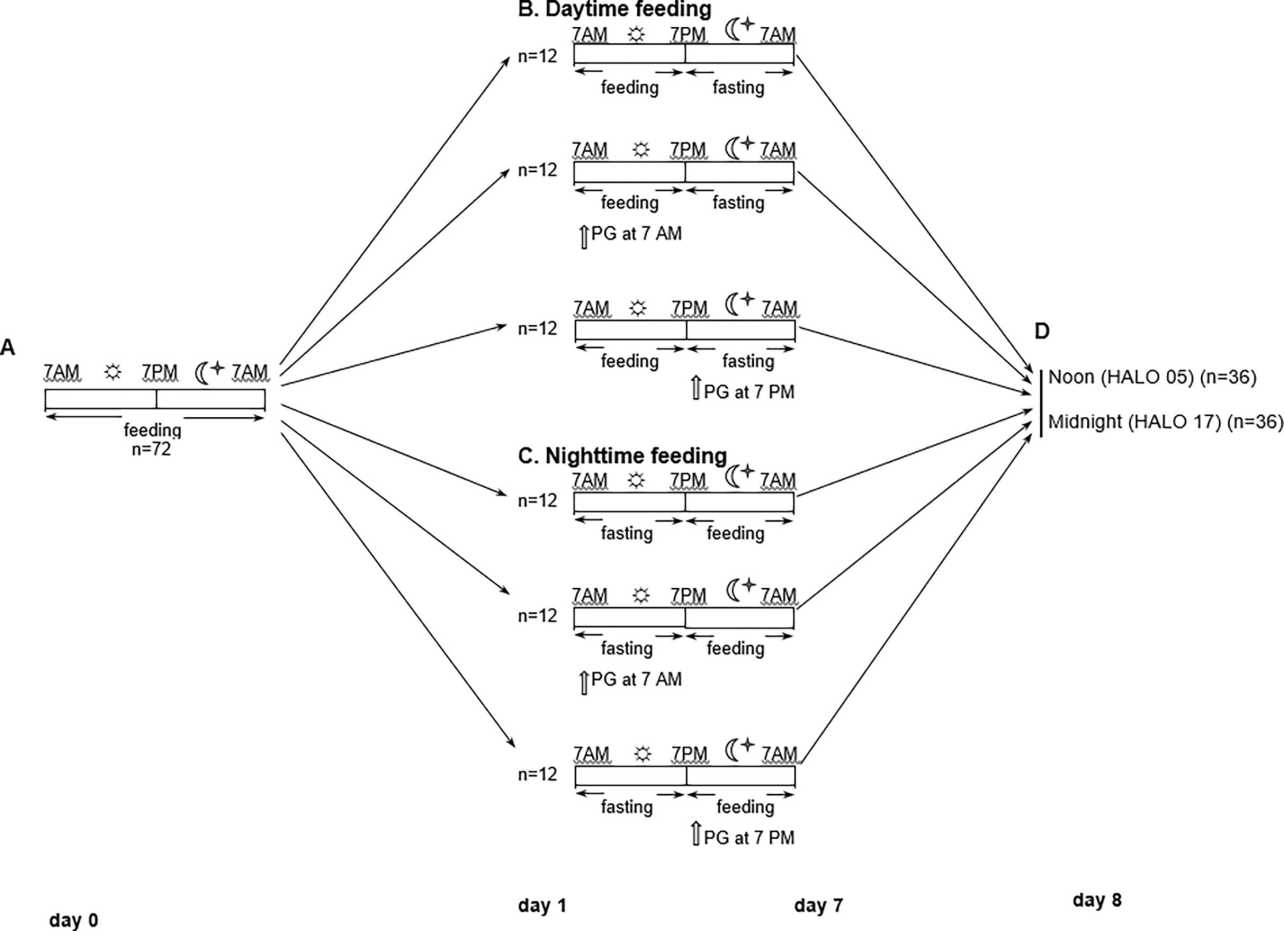
Experimental flowchart. Sun ☼ and Moon 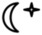 pictograms indicate the light and dark periods, respectively. Experimental schedule from Day 0 to Day 8: (a) mice entrained to a 12‐h light–dark cycle with ad libitum access to food and water. (b) Mice in RF groups. (c) Mice in NF groups. (d) Mice were sacrificed on Day 8 at noon (HALO 05) and midnight (HALO 17), and lung specimens and BAL samples were collected. Sacrifice for histopathological analysis was performed at HALO 05. White arrows indicate the time of PZ administration. HALO, hour after light onset.

We placed male BALB/c mice in single cages to avoid aggression and kept them following a light–dark cycle of a 12:12 pattern (lights on at 7 a.m.; lights off at 7 p.m.). From 4 weeks of age, the mice were housed individually, with unlimited access to food and water. The Ethics Committee of Poltava State Medical University approved the study. At 8 weeks of age, the mice were randomly divided into daytime feeding (RF) and nighttime feeding (NF) groups, with water provided ad libitum. Normally, the mice have increased activity and feed in the dark period. Thus, the daytime feeding is called reversed or inverted feeding. The RF group received food from 7 a.m. to 7 p.m., whereas the NF group received food from 7 p.m. to 7 a.m. (Xin et al., 2021). PZ was administered orally as an aqueous suspension of 40 μL at a dose of 20 mg/kg, either at 7 a.m. or 7 p.m., as previously described (Fedchenko et al., 2022). Each group consisted of 12 animals, and all manipulations during the dark phase were conducted under red light. The study design is shown in Figure 1.

On the eighth day of the feeding intervention, mice were euthanized by means of cervical dislocation at two different time points—noon (5 h after light onset or HALO 05) and midnight (HALO 17). The lungs were extracted, promptly frozen, and preserved at a temperature of −80°C for future use.

### 2.2 RNA preparation and quantitative reverse transcription PCR

For RNA extraction, lung tissue samples were collected from six mice at each time point. The RNeasy kit (QIAGEN), Cat. No. 74104, was used to extract total RNA from the lung tissue.

We generated single‐strand DNAs by reverse transcribing approximately 1 μg of total RNA from each sample using the QuantiTect® Reverse Transcription Kit (QIAGEN), Cat. No. 205313.

For SYBR Green‐based analysis, the QuantiTect® SYBR Green PCR Kit (QIAGEN), Cat. No. 204143, was used to amplify the cDNA equivalent of 50 ng of total RNA from each sample in the CFX96TM RealTime PCR Detection System (BIO‐RAD).

Data accuracy was ensured by analyzing each sample in duplicate. The gene expressions were detected as 2^−ΔCt^, and all values were normalized to the expression of the housekeeping gene β‐actin, which had weak circadian variation in the lung (Matsumura et al., 2014).

The specific primer sequences used for real‐time PCR are shown in Table 1. All the oligonucleotides were obtained from Metabion International AG (Germany).

**TABLE 1:** Primer sequences for mRNA measurement.

Gene	Primer sequences	References	ID number
*Arntl*	Forward ACATAGGACACCTCGCAGAA Reverse AACCATCGACTTCGTAGCGT	(Liu et al., 2007)	211116B056A01 211116B056B01
*Clock*	Forward CCTATCCTACCTTCGCCACACA Reverse TCCCGTGGAGCAACCTAGAT		211116B056C01 211116B056D01
*Nr1d1*	Forward CGTTCGCATCAATCGCAACC Reverse GATGTGGAGTAGGTGAGGTC		211116B056A03 211116B056B03
*Cry1*	Forward TTGCCTGTTTCCTGACTCGT Reverse GACAGCCACATCCAACTTCC		211116B056C03 211116B056D03
*Cry2*	Forward TCGGCTCAACATTGAACGAA Reverse GGGCCACTGGATAGTGCTCT		211116B056E03 211116B056F03
*Per1*	Forward CATGACTGCACTTCGGGAGC Reverse CTTGACACAGGCCAGAGCGTA		211116B056G03 211116B056H03
*Per2*	Forward GGCTTCACCATGCCTGTTGT Reverse GGAGTTATTTCGGAGGCAAGTGT		211116B056A04 211116B056B04
*Nfe2l2*	Forward CGCCGCCTCACCTCTGCTGCCAGTAG Reverse AGCTCATAATCCTTCTGTCG	(Ghosh et al., 2017)	211116B056C05 211116B056D05
*Pparg*	Forward CCAGAGCATGGTGCCTTCGCT Reverse CAGCAACCATTGGGTCAGCTC	(Illesca et al., 2019)	211116B056G04 211116B056H04
*Rela*	Forward GAGGTCTCTGGGGGTACCAT Reverse AAGGCTGCCTGGATCACTTC		211116B056A05 211116B056B05
*Cxcl5*	Forward TGCCCTACGGTGGAAGTCAT Reverse AGCTTTCTTTTTGTCACTGCCC	(Smith et al., 2017)	211116B056E05 211116B056F05
*β‐actin*	Forward ACTGCCGCATCCTCTTCCTC Reverse CTCCTGCTTGCTGATCCACATC	(Illesca et al., 2019)	211116B056E01 211116B056A02

### 2.3 Bronchoalveolar lavage (BAL)

On Day 8, mice were anesthetized using diethyl ether. The lungs were lavaged three times with 0.6 mL of 0.9% sodium chloride via the insertion of a tracheal tube (Mortola & Seifert, 2002). The lavage fluid was collected and centrifuged, and the supernatant was frozen at −80°C for subsequent cytokine analysis. The cells obtained from the BAL were resuspended in 1 mL of 0.9% sodium chloride solution, and the total cell count was determined using a count chamber. Glass slides were prepared and stained with Romanowsky‐Giemsa stain for performing differential cell counts.

### 2.4 BAL cytokines assay

We measured the concentration of interleukin‐6 (Cat. No. M600B), TNF‐alpha (Cat. No. MTA00B), and TGF‐beta 1 (Cat. No. DY1679) in the BAL fluid using kits from R&D Systems (Minneapolis MN). The absorbance was determined by LabLine‐026 (Labline).

### 2.5 Histological analysis

To perform histological analysis, the left lobe of each animal's lung was removed and inflated with 10% formalin after sacrifice. Paraffin‐embedded lung tissue 3 μm sections were stained with Hematoxylin and Eosin (H&E; Abcam, Cat. No. ab245880) for examination by histopathology. Morphological analyses were carried out using a light microscope Axio Lab.A1 (Carl Zeiss) and Zen 2.5 lite (blue edition) software. A blinded pathologist reviewed all lung sections using a special scoring system (Murakami et al., 2002).

To detect airway mucus, we used the periodic acid—Schiff (PAS) staining technique (Abcam, Cat. No. ab150680) and quantified PAS‐positive cells through a semiquantitative approach (Sundar, Ahmad, et al., 2015b; Yao et al., 2008).

We also conducted Mallory's trichrome staining (Abcam, Cat. No. 150686) and used the Ashcroft scoring system was used for fibrosis quantification (Ashcroft et al., 1988).

### 2.6 Statistical analysis

Descriptive statistics (M ± SD) was applied for the analysis of data using GraphPad Prism 5.0 software (San Diego, USA). The two‐way ANOVA and post hoc Bonferroni tests were employed to compare gene expression and cytokines concentration. The one‐way ANOVA test with post hoc tests and 2 × 2 table statistics, including Fisher's exact test, were used to analyze histopathological data and compare proportions. *p*‐values of <0.05 were considered statistically significant.

The study relied on the null hypothesis that RF, PZ, and the time of its administration did not influence the expression of clock, inflammatory, and metabolic genes in mouse lungs.

## 3 RESULTS

### 3.1 Changes in Per1, Per2, Cry1, Cry2, Clock, Arntl, and Nr1d1 mRNA after RF and PZ treatment in the mouse lungs

**FIGURE 2:**
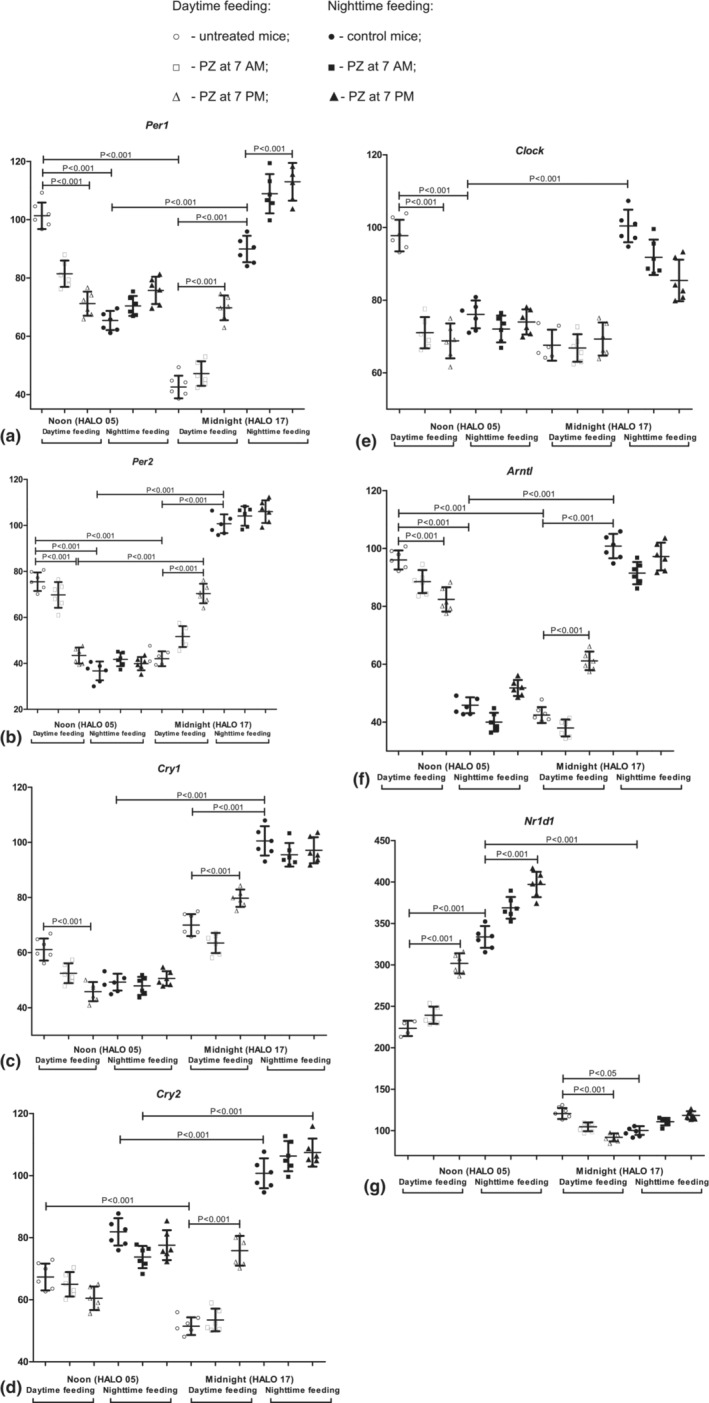
Diurnal changes in clock gene mRNA in mouse lung. Expression of mRNA: (a) *Per1*; (b) *Per2*; (c) *Cry1*; (d) *Cry2*; (e) *Clock*; (f) *Arntl*; and (g) *Nr1d1*. Horizontal lines show significant differences. *Y*‐axis—relative mRNA levels.

The levels of core clock genes' mRNAs during NF and after RF and PZ administration are shown in Figure 2. We observed statistically significant elevation of *Per1*, *Per2*, *Cry1*, *Cry2*, *Clock*, *and Arntl* mRNA at midnight than at noon in the lung tissues of NF mice. The level of *Nr1d1* mRNA was increased at noon. The core clock gene expression did not change after the oral intake of PZ at 7 a.m. At midnight, *Per1* mRNA was increased after PZ administration at 7 p.m. (*p* < 0.001).

RF influenced the core clock genes’ expression profile. *Per1*, *Per2*, *Clock*, and *Arntl* mRNA were decreased at midnight and increased at noon. At midnight, *Cry1* and *Cry2* mRNA were decreased in comparison with NF (*p* < 0.001). Also, we observed an increased *Nr1d1* mRNA at midnight with a decrease at noon (*p* < 0.001). There were no changes in core clock genes’ expressions after PZ at 7 a.m., excluding a slight decrease in *Clock* mRNA at noon. The treatment with PZ at 7 p.m. led to increased *Per1*, *Per2*, *Cry1*, *Cry2*, and *Arntl* mRNA at midnight. We also found PZ treatment at 7 p.m. reduced *Per1*, *Per2*, *Cry1*, *Clock*, *and Arntl* mRNA as well as induced *Nr1d1* mRNA at noon. Taken together, these findings reflect a normalization of the core clock mRNA profile under the influence of PZ treatment at 7 p.m.

### 3.2 Changes in Nfe2l2, Pparg, Rela, and Cxcl5 mRNA after RF and PZ treatment in the lungs of mice

**FIGURE 3:**
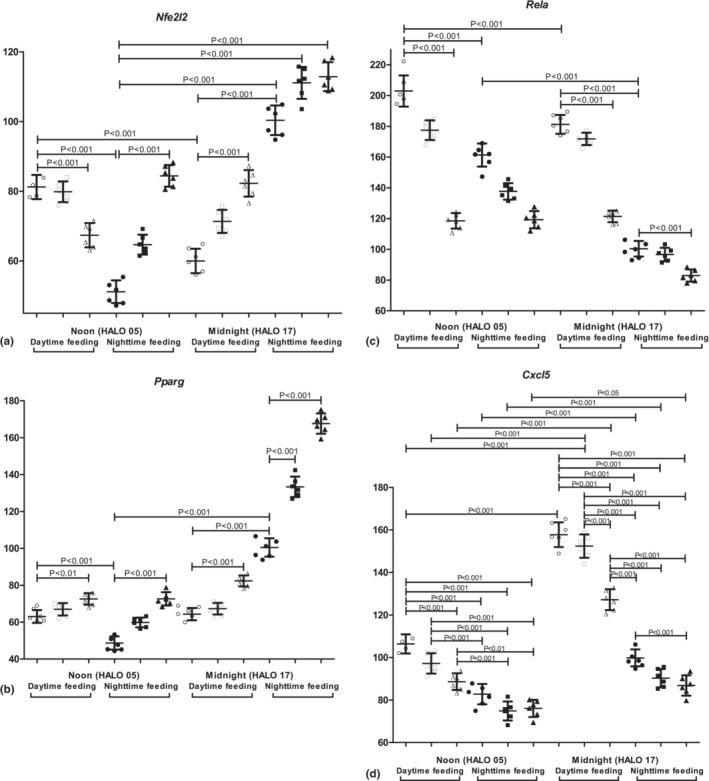
Circadian changes in mRNA transcription of inflammation‐/metabolism‐related genes in the lungs of mice. Expression of mRNA: (a) *Nfe2l2*; (b) *Pparg*; (c) *Rela*; and (d) *Cxcl5*. Significant differences are shown by the horizontal line. *Y*‐axis—relative mRNA levels.

During NF, we found the increased levels of *Nfe2l2*, *Pparg*, and *Cxcl5* at midnight in comparison with noon expression. In contrast, *Rela* mRNA was significantly reduced at midnight (*p* < .01; Figure 3).

In RF mice, we observed the reversed profile of *Nfe2l2* gene expression with the prevalence of noon expression over midnight expression. In general, the feeding intervention led to a decrease in *Nfe2l2* expression. We also found the loss of diurnal variation in *Pparg* expression in these animals. In turn, *Rela* and *Cxcl5* mRNAs were reduced at midnight and noon compared with NF mice. The level of *Pparg* mRNA had a statistically significant reduction at midnight. PZ treatment of NF mice at 7 a.m. caused an increase in *Pparg* mRNA and a decrease in *Cxcl5* mRNA at midnight.

PZ administration at 7 a.m. to nighttime feeding mice increased *Pparg* and decreased *Cxcl5* expression at midnight expression. On the contrary, PZ treatment at 7 p.m. elevated *Nfe2l2* expression at noon and *Pparg*—at midnight, as well as decreased *Rela* expression at midnight and noon. We also observed a decrease in *Cxcl5* mRNA at midnight. There were no statistically significant differences in mRNAs concentrations after PZ treatment at 7 a.m. in RF mice. Administration of PZ at 7 p.m. induced midnight expression of *Nfe2l2* and decreased noon expression, as well as increasing midnight and noon expression of *Pparg*. PZ treatment at 7 p.m. decreased *Rela* and *Cxcl5* mRNA at midnight and noon.

### 3.3 Changes in inflammatory cell influx in BAL fluid after RF and treatment

We observed the prevalence of total cells and neutrophil count in NF mice at midnight. PZ treatment at 7 a.m. reduced neutrophil count at midnight, and macrophages—at noon as well as increased neutrophil count at noon. PZ treatment at 7 p.m. elevated macrophages and neutrophil count at midnight as well as neutrophils at noon. RF led to the elevation of total cells, macrophages, and lymphocyte count at midnight, accompanied by the reduction in neutrophil count at midnight and noon.

**FIGURE 4:**
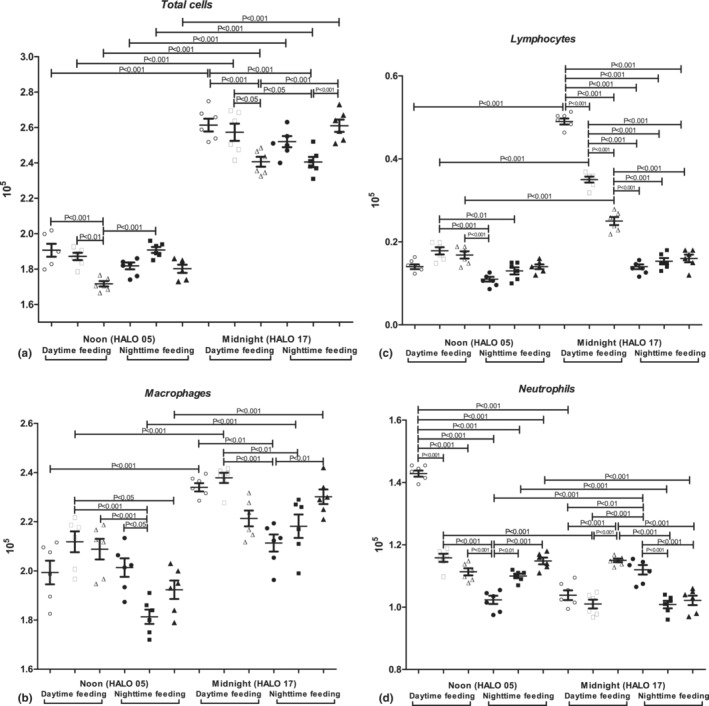
Influence of RF and PZ treatment on inflammatory cell influx in mouse BAL: (a) total cells; (b) macrophages; (c) lymphocytes; and (d) neutrophils.

PZ treatment at 7 a.m. reduced lymphocyte count at midnight and neutrophil count at noon. We also found PZ intake at 7 p.m. statistically significantly decreased total cells, macrophages, and lymphocyte count and increased neutrophil count at midnight, as well as decreased total cells and neutrophil count at noon (Figure 4).

### 3.4 Changes in cytokine release in BAL fluid after RF and PZ treatment

**FIGURE 5:**
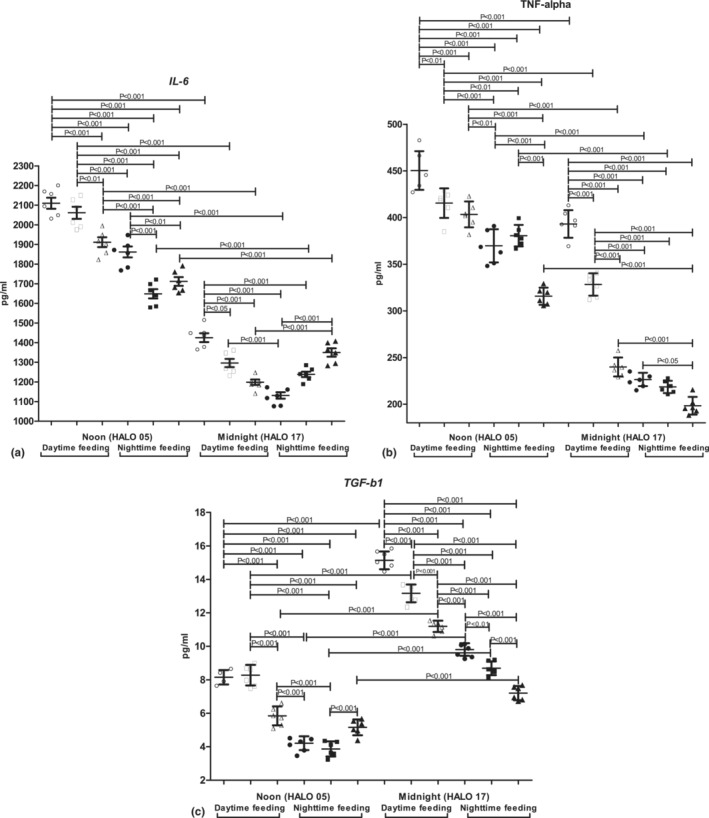
Changes in mouse BAL cytokines levels after RF and PZ treatment: (a) IL‐6; (b) TNF‐alpha; (c) TGF‐beta 1.

At nighttime, there was a significant diurnal variation of BAL cytokines concentration with the pro‐inflammatory IL‐6 and TNF‐alpha prevalence at noon and an increased concentration of anti‐inflammatory TGF‐beta 1 at midnight (Figure 5).

There were significant changes in cytokines concentrations in BAL: a significant increase in IL‐6, TNF‐alpha, and TGF‐beta 1 at all time points.

PZ administration at 7 a.m. to nighttime feeding mice decreased a level of TGF‐beta 1 at midnight and IL‐6 at noon. In RF mice, there was a reduction in IL‐6, TNF‐alpha, and TGF‐beta at midnight, and TNF‐alpha at noon.

PZ treatment at 7 p.m. increased the level of IL‐6 at midnight and decreased at noon compared with untreated animals. PZ intake at 7 p.m. reduced TNF‐alpha concentration at noon in comparison with PZ treatment at 7 a.m. and decreased at midnight and noon compared with untreated animals. A statistically significant reduction in TGF‐beta 1 concentration at midnight in comparison with animals treated with PZ at 7 a.m. as well as with untreated mice. In RF mice, PZ administration led to a decrease in IL‐6 concentration at noon compared with animals treated by PZ at 7 a.m. as well as to the decrease at all time points compared with the untreated mice. Administration of PZ at 7 p.m. reduced the TNF‐alpha and TGF‐beta 1 concentration at all time points in comparison with animals treated by PZ at 7 a.m. and untreated mice (with an exception for TNF‐alpha at noon in comparison with morning administration of PZ).

### 3.5 The changes in mouse lung morphology after RF and PZ treatment

**FIGURE 6:**
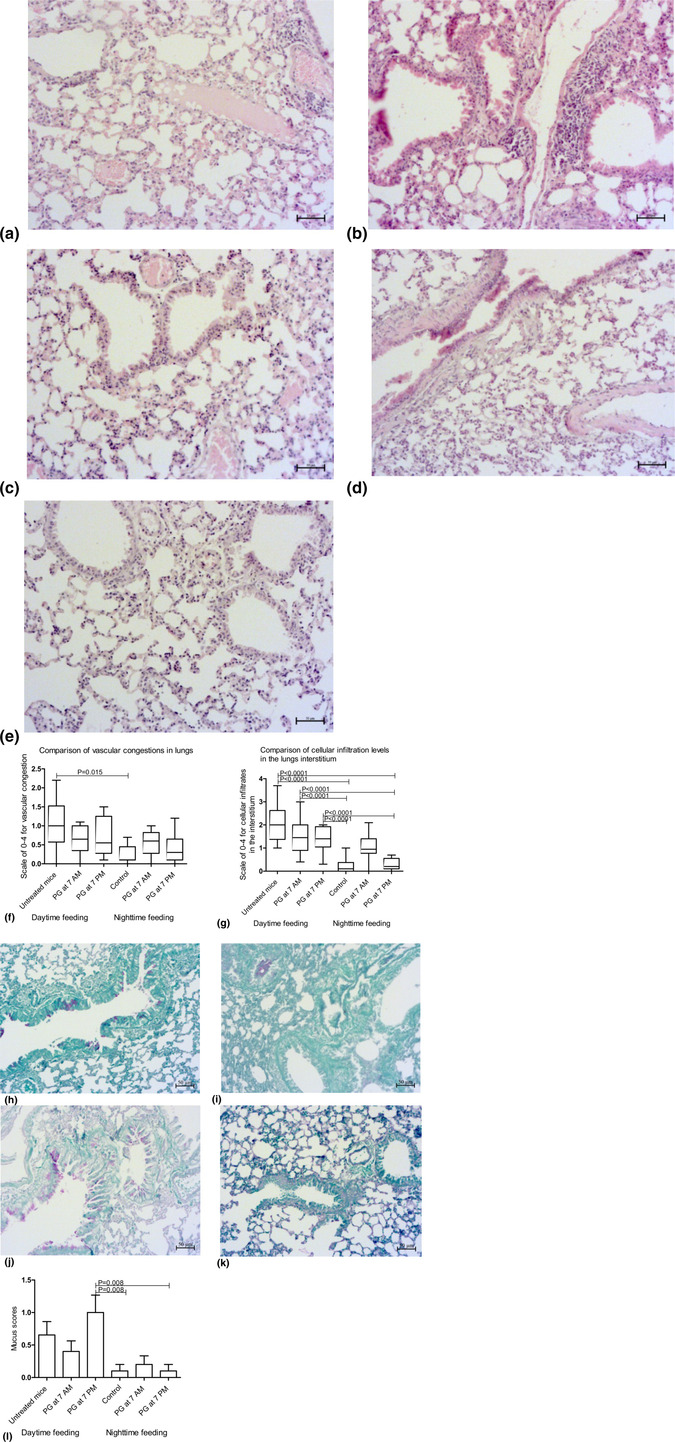
Lung tissue changes and histopathological scores after RF and PZ treatment (H&E). *S*cale bars 50 μm. (a) Vascular congestion (asterisks) and cellular infiltrates (white arrows) in RF *u*ntreated mice; (b) marked cellular infiltrates (black arrows) in RF *PZ* 7 a.m. mice; (c) absent of visual changes in vascular congestion in RF *PZ* 7 p.m. mice; (d) nighttime feeding PZ 7 p.m. and (e) control groups demonstrate similar minimal levels of vascular congestion and cellular infiltrations of lung interstitium; (f) Comparisons of mean scores of vascular congestion and (g) cellular infiltrates: *P*‐value calculated by Kruskal–Wallis and Dunn's post hoc test; (h) mucus overproduction mice (PAS staining) in the bronchial epithelium of RF untreated; (i) PAS reactivity in bronchial cast after PZ intake at 7 a.m.; (j) mucus overproduction in bronchial epithelium of RF *PZ* 7 p.m. mice; (k) PAS reactivity mostly absent in goblet cells of control mice; and (l) comparisons of mean mucus scores: *P*‐value calculated by Kruskal–Wallis and Dunn's post hoc test.

In RF‐untreated animals, we found a significant elevation of vascular congestion and interstitial cellular infiltrates (Figure 6a). PZ intake at 7 a.m. and at 7 p.m. reduced levels of vascular congestion and cell infiltration but did not reach statistical significance (Figure 6b–g). In RF‐untreated animals, some mucus overproduction was observed in the bronchial epithelium (Figure 6h). PZ treatment at 7 a.m. reduced mucus production but did not reach statistical significance (Figure 6i,l). PZ treatment at 7 p.m. elevated mucus production, whose mean level statistically prevailed over control groups (Figure 6j,l). There were no statistically significant collagen depositions in lung tissue in all experimental or control groups (data not shown).

## 4 DISCUSSION

The circadian rhythm is an essential regulatory mechanism orchestrating physiological processes and cellular metabolism in living organisms. This mechanism is based on complicated molecular factors driven by extrinsic and intrinsic stimuli (Bass & Takahashi, 2010). In the body, there is a strong hierarchy of the circadian rhythm from the suprachiasmatic nucleus to the tissue/cellular oscillators. These oscillators rely on the transcriptional and translational feedbacks regulating the core clock and related genes (Takahashi, 2017). The genetic control of circadian rhythms is provided by the core clock genes consisting of *Clock*, *Npas 2*, *Arntl*, *Per1*/*Per2*, *Cry1*/*Cry2*, and *Nr1d1/2*), and *Nr1/2/3*. The protein products of these genes take part in the regulation of more than 80% of genes expressed throughout the body (Mure et al., 2018; Zhang, Lahens, et al., 2014).

While environmental light and dark changes are the main conventional input for the circadian rhythm regulation, other lifestyle factors also influence it. These factors include food consumption, night‐ and evening‐time eating, exercise, temperature, and stress (Güldür & Otlu, 2017; Panda et al., 2002). A changed time of eating (such as the shifting of food consumption to evening or night) is associated with sleeping disorders, weight gain, and physical activity reduction in a healthy person (Yahia et al., 2017). The evening chronotype is also associated with increased sleep apnea (Lucassen et al., 2013). Night eating syndrome was described in patients suffering from being overweight. This is a type of eating disorder related to eating after dinner and being awake at night (Salman & Kabir, 2022). At the same time, the obese patients had long morning fasting (O'Connor et al., 2022).

Patients with metabolic syndrome and type 2 diabetes had a high cardio‐metabolic risk and nighttime eating patterns. Moreover, nighttime work and other lifestyle disorders might induce glucose intolerance and diabetes (Ha & Song, 2019; Quist et al., 2021; Shan et al., 2018).

In previous studies, it has been shown that an imbalance in the circadian rhythm led to immune disorders and allergy promotion due to different pathways, including epithelial dysfunction (Nakao, 2020). Circadian disruption is involved in various pathologies: gluco‐ and mineralocorticoids nighttime hypertension; shift work intolerance; peptic ulcer disease; kidney failure; nocturia; asthma; cancer; hand, foot, and mouth diseases and ICU outcome (Smolensky et al., 2016).

Recently chronobiological aspects of chronic airway diseases have been under active investigation. The disturbance of the circadian rhythm might affect lung cellular and molecular physiology (Sundar, Yao, et al., 2015c). The worsening of asthma and COPD clinical symptoms (cough, shortness of breathing, decrease in respiratory volumes, increased mucus production and sleeping disorders), and changed therapeutic efficacy to bronchodilators and steroids are observed at nighttime (Agusti et al., 2011; Casale & Pasqualetti, 1997; Chinnapaiyan et al., 2020; Gebel et al., 2006; Sundar et al., 2018; Tsai et al., 2007; Yao & Rahman, 2011). Lung viral infections remodel pulmonary clock functions in COPD and asthma (Ehlers et al., 2018; Sundar, Ahmad, et al., 2015b).

There are no recent sufficient data on how late‐evening and nighttime eating influence physiological functions and the molecular clock in the lung. RF might be an effective rodent model for the investigation of the time‐of‐day eating variations. This experimental model breaks the peripheral molecular clock in the mouse heart, liver, and kidney (Fedchenko et al., 2022; Izmailova et al., 2022; Oyama et al., 2014; Yamamura et al., 2010).

We demonstrated that the RF led to different expression patterns with decreased levels of *Per1*, *Per2*, *Clock*, and *Arntl* mRNA at midnight and an increase at noon. At midnight, there was the level of *Cry1* and *Cry2* mRNAs was decreased in the lungs of RF mice. *Nr1d1* mRNA was increased at midnight and decreased at noon. These data showed that RF reprogrammed the peripheral lung clock. Our observation correlated with other references that the time of food consumption was a powerful factor affecting the circadian rhythm in metabolically active organs and tissues (Bilu et al., 2022; Glad et al., 2011; Kim et al., 2023; Pickel & Sung, 2020; Woodie et al., 2022). For lung circadian molecular clock gene expression, such influence was described for tobacco smoking (Choukrallah et al., 2020; Khan et al., 2019; Numaguchi et al., 2016). In this context, we have highlighted the suppression of *Nr1d1* expression that might be parallel with increased inflammation and inflammation‐related cytokines concentrations (Lechasseur et al., 2017).

Simultaneously, we investigated the expression of several inflammation‐ (*Rela* and *Cxcl5*) and metabolism‐related genes (*Nfe2l2*, *Pparg*). The RF reversed the profile of *Nfe2l2* gene expression with the prevalence of noon expression over midnight expression accompanied by the loss of diurnal variation in *Pparg* expression. The transcriptional factor NRF2 contributed to the regulation of lung inflammation (Kobayashi et al., 2013; Qin et al., 2015) and fibrosis (Wang et al., 2022). NRF2 might procure the scavenge of apoptotic neutrophils by alveolar macrophages (Reddy et al., 2022), regulate free radical oxidation, possess antioxidant activity protecting cells and subcellular compartments, and eliminate reactive oxygen species (Kovac et al., 2015).

*Nfe2l2* knockout promotes lung inflammation and injury (Cho et al., 2004; Reddy et al., 2009) and increases the levels of inflammatory cells in BAL after pollutant lung damage (Sehsah et al., 2019). NRF2 increases PGC‐1 alpha deacetylation and alleviates chromium‐induced lung damage (Han et al., 2019). On the contrary, activated PGC‐1 alpha cooperates with a variety of transcriptional factors, including NRF2 and PPARs (Baar, 2004; Bost & Kaminski, 2019; Chambers & Wingert, 2020).

PPARG realizes anti‐inflammatory and antioxidative actions, inducing *Nfe2l2* expression (Li, Liu, Feng, et al., 2022). PPARG regulates alveolar macrophage (AM) functions. AMs express a very high level of PPARG mRNA and protein (Reddy et al., 2004), neutrophil and eosinophil functions (Kintscher et al., 2000; Ueki et al., 2004), and allergic airway inflammation (Trifilieff et al., 2003). Thus, the reversed profile of *Nfe2l2* expression and the loss of diurnal variation in *Pparg* expression might provoke pro‐inflammatory and dysregulating processes in the lungs of RF mice.

A remarkable increase was observed in the midnight and noon expression of *Nfe2l2* and *Cxcl5* mRNA with pro‐inflammatory properties. NFκB axis has great importance for lung immunometabolism and injury (Mahung et al., 2022; Soto et al., 2020; Tang et al., 2018). NFκB through TNF‐alpha can stimulate cells to upregulate several pro‐inflammatory mediators such as neutrophilic chemokine CXCL5 (Kuret et al., 2022). Diurnal expression of CXCL5 was found in club cells, and this expression was regulated by the circadian rhythm. Elevated expression of CXCL5 due to the deletion of BMAL alleviated LPS‐induced inflammation (Gibbs et al., 2009). Such abrogation of BMAL activity decreases the resistance to respiratory viral infections, advantages the viral entry and replication, and promotes injury of bronchoalveolar epithelium and mucus production (Ehlers et al., 2018).

In our investigation, the RF led to the increase in total cells, macrophages, and lymphocyte count in BAL at midnight and neutrophil count at noon, accompanied by a significant increase in vascular congestion and cellular infiltrates in mouse lungs. These findings went in parallel with the enhanced expression of *Rela* and *Cxcl5* mRNA. Elevated BAL cellularity was accompanied by a statistically significant increase in IL‐6, TNF‐alpha, and TGF‐beta 1 at midnight and noon.

Taken together, our findings suggest that RF causes the disturbance of male mouse lung diurnal expression of core clock genes, accompanied by the elevation of *Rela* and *Cxcl5* mRNAs and diminution of *Nfe2l2* and *Pparg* mRNAs. These impaired gene regulations increase low‐grade inflammation and possible metabolic disorders in lung tissue.

We investigated the ability of PZ to influence the mRNAs level of core clock genes and inflammation‐ and metabolism‐related gene disturbance by RF in mouse lungs. The influence of PZ and rosiglitazone on the clock gene expression was shown recently for mouse liver and kidney tissue (Fedchenko et al., 2022; Izmailova et al., 2022; Ribas‐Latre et al., 2019). New data were obtained about the role of PPARs and PPARG in lung chronobiology and chronotherapy (Nosal et al., 2020; Paudel et al., 2021; Sundar et al., 2018). Moreover, PPARG agonists have a therapeutic potential for pulmonary diseases as a modulator of inflammation induced by different stimuli, including bacteria and viruses (Carvalho et al., 2021).

We investigated the efficacy of PPARG agonist PZ administration at two time points—7 a.m. and 7 p.m. PZ administration at 7 a.m. did not affect diurnal core genes' expressions significantly as well as the levels of inflammation‐ and metabolism‐related genes mRNAs in RF mice. We also observed only a decrease in BAL neutrophil count and IL‐6, TNF‐alpha, and TGF‐beta 1 levels without statistically significant influence on lung morphology.

PZ treatment at 7 p.m. led to the restoration of diurnal expression of clock genes, including *Nr1d1*. REV‐ERB alpha is the product of *Nr1d1* and operates as one of the most important repressors of circadian regulation loops. REV‐ERB alpha interacts with specific DNA sites into clock gene sequences as well as other metabolism‐ and inflammation‐related genes, the patterns of these interactions are specific for different tissues (Butler & Burris, 2015; Zhang et al., 2015). REV‐ERB alpha might possess as a target for new therapies in chronic airway pathologies (Kojetin & Burris, 2014; Solt et al., 2012). Recent results have shown that REV‐ERB alpha might be induced by PPARG activation and controlled NFκB‐driven pro‐inflammatory genes, for example, IL‐6 (Laitinen et al., 2005).

In parallel to these data, PZ treatment at 7 p.m. induced *Nfe2l2* and *Pparg* and decreased *Rela* and *Cxcl5* expressions. *Nfe2l2* and *Pparg* have a functional agonism in the regulation of inflammation and metabolism. Moreover, PPARG regulates NRF2 activation (Gao et al., 2018; Li, Peng, Feng, et al., 2022), and the two factors inhibit NFκB‐mediated inflammation (Chen & Maltagliati, 2018). Inhibition of *Rela* might decrease *Cxcl5* expressions because *Cxcl5* is the direct NFκB target gene (Guan et al., 2016).

At the same time, PZ treatment at 7 p.m. decreases cell influx in BAL—macrophages and lymphocytes at different time points as well as IL‐6, TNF‐alpha, and TGF‐beta 1. During the inflammation, PPARG alleviates the activation and DNA‐binding activity of NFκB due to the repression of IκB‐alpha degradation. This repression is achieved by the activation of the IκB kinase or by the interaction of PPARG with the NFκB p50/NFκB p65 dimer (Zhang, Hu, et al., 2014; Zhu et al., 2016). Usually, between NFκB‐driven cytokines (IL‐6 and TNF‐alpha) and the TGF‐beta 1/Smad pathway, there is an antagonistic interaction but some dysfunctions might occur (Garg et al., 2022). Therefore, this observation needs further special investigation.

**FIGURE 7:**
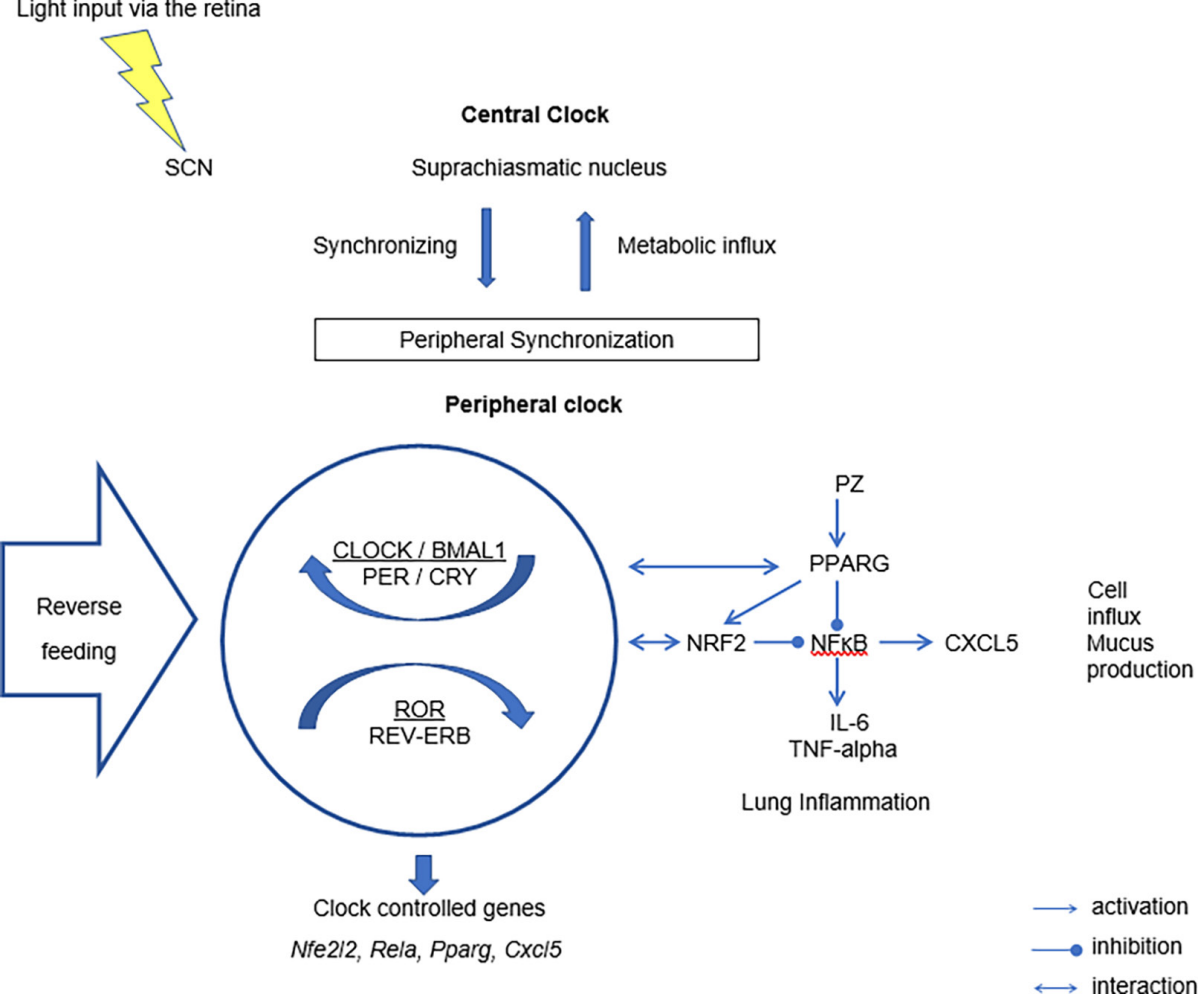
Light–dark changes are received by special light‐sensitive receptors in the retina and induced inputs to SCN, which acted as a central pacemaker of the circadian clock. In route SCN generals neurohumoral outputs to the tissue‐specific peripheral clocks (Dibner et al., 2010). A circle shows the core and stabilization loops of the core circadian oscillator. Tissue‐specific circadian transcription is regulated by REV‐ERB and retinoic acid receptor‐related orphan receptors (RORs), which interacted with specific response elements of the clock (including Arntl) and clock‐controlled genes. Peripheral oscillators might have taken away from the SCN control and developed peripheral rhythm due to eating or fasting (Damiola et al., 2000; Kim et al., 2022). RF induced the diurnal disruption of the core clock gene and inflammation‐/metabolism‐related gene expression pattern. Impaired gene expression increased the activity of the NFκB pathway and decreased NRF2/PPARG. PZ activated PPARG with consequent suppression of the NFκB pathway and decreased concentration of pro‐inflammatory cytokines IL‐6, TNF‐alpha, and cell influx in BAL.

In Figure 7, we summarized our findings and suggested the PZ activity during RF. In brief, RF disrupted the diurnal pattern of the core clock gene and inflammation‐ /metabolism‐related gene expression with enhancement of the NFκB pathway. PZ administration increased the expression of *Nfe2l2* and *Pparg* mRNA and alleviated the level of IL‐6, TNF‐alpha, and cell influx in BAL.

By using the same model of RF and PZ administration in male mice, we can compare the effects of such interventions in the three metabolic active organs—liver, kidney, and lung (Fedchenko et al., 2022; Izmailova et al., 2022).

RF induced a tissue‐unspecific disruption of the circadian expression pattern of core clock genes decreasing *Per1*, *Per2*, *Cry1*, *Cry2*, *Clock*, and *Arntl* with increased *Nr1d1* mRNAs at midnight. In contrast, we observed the differences in *Cxcl5* mRNA expression in mouse liver and lung tissue. RF induced a significant reduction in *Cxcl5* mRNA at midnight and noon.

PZ had a similar tissue‐unspecific effect, repaired the diurnal clock gene expression, and alleviated pro‐inflammatory factors as well as increased anti‐inflammatory potency with the maximum activity at 7 p.m. versus 7 a.m.

This study might be limited by the extra lung effects of RF and PZ. The duration of the recent animal model might be insufficient to develop more significant changes in lung tissue. Additional investigations are needed into TGF‐beta1‐related processes and mucus production during RF and PZ treatment are needed. Clock gene expression was analyzed in lung tissue homogenates. The changes in the cellular composition in lung tissue (e.g., recruitment of inflammatory cells, as shown) may affect the values for total clock gene expression in the tissue.

Further studies are needed to investigate chronic airway diseases and their interactions with the lung peripheral clock as well as the therapeutic potency of PPARG agonists. Additional experiments are needed with several time points (not only noon and midnight) to estimate circadian parameters (amplitude, MESOR, or phase) as well as with a model of prolongated (14 and more days) RF.

## 5 CONCLUSIONS

We concluded that the RF disrupted the lung diurnal expression of core clock genes, alleviating *Nfe2l2* and *Pparg*, and exaggerating *Rela* and *Cxcl5* expression accompanied by the elevation of IL‐6, TNF‐alpha concentrations as well as cellularity in BAL.

PZ treatment repaired the diurnal clock gene expression, increased *Nfe2l2* and *Pparg* mRNAs, and alleviated *Rela*, *Cxcl5* mRNAs, and IL‐6, TNF‐alpha, and cellularity in BAL. PZ treatment had a significantly higher chronopharmacological activity at 7 p.m. than at 7 a.m.

## AUTHOR CONTRIBUTIONS

Oksana Shlykova contributed to investigation, methodology, and visualization. Olga Izmailova contributed to investigation, methodology, data curation, and statistical analysis. Alina Kabaliei and Vitalina Palchyk contributed to investigation and visualization. Viktoriya Shynkevych contributed to investigation, visualization, and writing—original draft. Igor Kaidashev contributed to project administration, methodology, data curation, visualization, writing—original draft and writing—review and editing.

## FUNDING INFORMATION

The study was supported by grants No. 0120U101166 “The study of the pathogenetic role of the circadian molecular clock in the development of metabolic diseases and systemic inflammation and the development of treatment methods aimed at these processes” and No. 0122U201686 “Development of methods for treatment and prevention of pulmonary fibrosis by activation of PPAR‐gamma receptors” funded by the Ministry of Public Health of Ukraine.

## CONFLICT OF INTEREST STATEMENT

The authors declared no competing interests.

## ETHICS STATEMENT

All experimental procedures were ethically approved by the Committee on Bioethics and Ethical Issues of Poltava State Medical University.

## Data Availability

The datasets generated during and/or analyzed during the current study are available from the corresponding author upon reasonable request.
